# Renal and Inflammatory Proteins as Biomarkers of Diabetic Kidney Disease and Lupus Nephritis

**DOI:** 10.1155/2022/5631099

**Published:** 2022-03-20

**Authors:** Nathan H. Johnson, Robert W. Keane, Juan Pablo de Rivero Vaccari

**Affiliations:** ^1^Department of Physiology and Biophysics, University of Miami Miller School of Medicine, Miami, FL, USA; ^2^Department of Neurological Surgery and The Miami Project to Cure Paralysis, University of Miami Miller School of Medicine, Miami, FL, USA

## Abstract

Current methods for differentiation of kidney disease types are unspecific and may be invasive. Thus, there is a need for development of new biomarkers of kidney disorders that are specific and less invasive. In this study, we analyzed serum samples of diabetic kidney disease (DKD) and lupus nephritis (LN) patients to identify biomarkers of these two disorders. Serum samples were analyzed by Simple Plex assays. We calculated the area under the curve (AUC) as well as receiver operating characteristics (ROC) to obtain the sensitivity and specificity and other biomarker-related variables of apoptosis-associated speck-like protein containing a caspase recruitment domain (ASC), interleukin- (IL-) 18, Lipocalin-2/NGAL, epidermal growth factor (EGF), u-Plasminogen Activator (uPA), and C-reactive protein (CRP) as potential biomarkers. Protein levels of ASC, IL-18, EGF, and Lipocalin-2/NGAL were higher in DKD and LN patients when compared to controls, whereas only uPA was elevated in DKD patients and CRP in LN patients. As determined by the AUC, of the six analytes studied, EGF (AUC = 0.9935), Lipocalin-2/NGAL (0.9554), ASC (0.7666), and uPA (0.7522) are reliable biomarkers of DKD, whereas EGF (1.000), Lipocalin-2/NGAL (0.9412), uPA (0.7443), and IL-18 (0.7384) are more reliable for LN. The biomarkers analyzed can differentiate between healthy and affected individuals. However, there was no difference between the levels of these biomarkers in DKD vs LN. Thus, although these biomarkers cannot be used to categorize patients between DKD and LN, they are useful as biomarkers of renal pathology.

## 1. Introduction

Kidney disease is a major health concern impacting more than 750 million people throughout the world and represents a significant burden to patients and health care systems [1]. Kidney disease is a generalized term representing an assortment of disorders impacting the renal system, each with complex and often uniquely distinctive pathologies [2]. Currently, the most widely used method for the diagnosis of kidney disease is through the measurement of the glomerular filtration rate (GFR) [2, 3]. Although long considered a “golden standard” for the detection of kidney disorders, a patient's GFR is typically determined by measuring the concentrations of kidney filtrates in blood plasma or in urine [3]. Since the GFR is the collective product of millions of glomeruli working concurrently, a patient's true GFR is often very difficult to establish [3]. Clinicians traditionally calculate an estimated glomerular filtration rate (eGFR) through measurement of serum creatinine and blood urea nitrogen (BUN) concentrations [2, 3]. Urinalysis may also be used to calculate a measured glomerular filtration rate (mGFR) by quantifying clearance of a readily filtered, exogenous substance such as inulin. Urinalysis is also used to detect proteinuria and to determine the albumin levels [2, 3]. Unfortunately, these methods traditionally used to determine GFR are nonspecific to the underlying cause of pathology and are often not significantly altered until much later in disease progression [2–4].

Of the kidney diseases, diabetic kidney disease (DKD) and lupus nephritis (LN) are two chronic disorders that are typically not detected by altered GFR or proteinuria until much later in their development [4, 5]. In diabetic patients, DKD diagnosis is traditionally made when one or more of the following signs is present over a span of three months: a decreased eGFR (<60 ml/min/1.73 m^2^), an increase in daily albumin excretion in urine (≥30 mg), or an increase in the urinary albumin/creatinine ratio (≥30 mg g^−1^) [5]. In patients suffering from systemic lupus erythematosus (SLE), the development of LN is determined by increased protein in urine (>0.5 g daily), from the presence of cellular casts in urine, or from an increased urinary creatinine/protein level (>0.5) [6]. Additionally, LN may be diagnosed through renal biopsy. However, this method is invasive and potentially hazardous [4, 6]. Although DKD and LN are two different disorders, they have shared pathological attributes that are seen in almost every kidney disorder. Specifically, both disorders are known to cause increased expression of inflammatory proteins within the kidneys through activation of the inflammasome [7, 8].

The inflammasome is a multiprotein structure that allows for the assembly of inflammatory caspases and subsequent activation of cytokines to signal inflammation and cell death in native and neighboring cells [7, 9]. In cases of acute kidney injury and chronic kidney disease, the inflammasome has demonstrated a significant role in pathological development, as studies interfering with inflammasome activity have shown reduction in or regulation of pathological progression [8]. In DKD, studies have shown increased nucleotide binding domain- (NOD-) like receptor protein 3 (NLRP3) and absent in melanoma 2 protein (AIM2) expression within the kidneys and increased expression of the inflammatory cytokine interleukin- (IL-) 1*β* in serum [7]. In LN, studies have shown increased expression of NLRP3, caspase 1, and IL-1*β* in tissue collected by kidney biopsies and in podocytes [7, 10]. Thus, these studies show that the inflammasome is a promising target for the development of future therapeutics for renal diseases. Moreover, inflammasome proteins have been shown to be biomarkers of the inflammatory response associated with diagnosis of traumatic brain injury [11–14], stroke [15], depression [16], multiple sclerosis [17], mild cognitive impairment [18], Alzheimer's disease [18], nonalcoholic steatohepatitis [19], psoriasis [20], and age-related macular degeneration [21].

Identification of biomarkers involved in renal pathology has the potential to be used to monitor disease progression in patients newly diagnosed with diabetes or lupus so that more personalized treatment strategies may be utilized. Moreover, inflammasome proteins can also be used as theragnostic biomarkers to identify whether patients are responding positively or negatively to different treatment strategies. The purpose of this study was to investigate biomarkers of renal disease independently of whether the disease has an autoimmune origin or a chronic disease known to affect the kidneys. Moreover, considering the multifactorial aspects that contribute to renal diseases, in this study, we analyzed a combination of kidney disease-associated proteins (Lipocalin/NGAL, EGF and uPA), as well as proteins associated with inflammasome activity (ASC and IL-18) and general inflammation (CRP) with the aim of determining their reliability as potential biomarkers of kidney disease, specifically, DKD and LN.

## 2. Materials and Methods

### 2.1. Participants

Serum from patients with DKD and LN (Table 1) and age-matched controls were obtained from BiolVT (Hicksville, NY). Donor informed consent was provided by those participating in the study Prospective Collection of Samples for Research funded by SeraTrials, LLC. with IRB number 20170439. Age-matched control samples were obtained from patients with no medical diagnosis of any disease with a median of 50 years old, a mean of 49.02, and a range between 21 and 101. Patients in the DKD group had a median age of 62 years old and a range between 21 and 90, and those with LN had a median age of 43.5 and a range between 19 and 75. Patients in the DKD cohort consisted of 17 Caucasians, 10 of African descent, and 4 of other race, whereas in the LN group there were 17 Caucasians, 11 of African descent, and 4 of other races. Patients with DKD presented the following comorbidities: hypertension (23 patients), hypercholesterolemia, (6 patients) hypothyroidism (8 patients), gastroesophageal reflux disease (7 patients), and coronary artery disease (7 patients), whereas patients in the LN group presented with Hypertension (6 patients), gastroesophageal reflux disease (2 patients), and coronary artery disease (3 patients).

DKD patients were characterized by a cohort representing 4 of the 5 stages of the disease as determined by the eGFR in ml/min/1.73 m^2^: 2 (guarded: 60-89), 3 (elevated: 30-59), 4 (high: 15-29), and 5 (severe: <15 or dialysis) [22]. Moreover, LN patients were in part diagnosed by kidney biopsy representing the following classifications: 2 (mesangial proliferative LN), 3 (focal LN affecting less than 50% of glumeruli), 4 (diffuse LN affecting more than 50% of glumeruli), and 5 (advanced sclerosing LN) [23].

In summary, in this proof-of-concept study, the inclusion/exclusion criteria were characterized by including patients with a positive diagnosis of either DKD or LN without an exclusion for age, race, or disease stage. Patients were included in the DKD cohort regardless of whether patients presented with either type I or type II diabetes. Due to the effects of DKD and LN on the renal system, patients with hypertension with a positive diagnosis for either DKD or LN were also included in the study. However, not having a diagnosis of HTN was not considered an excluding factor.

### 2.2. Simple Plex Assay

Concentration of inflammasome proteins (ASC and IL-18) and indicators of renal function (uPA, EGF, and Lipocalin/NGAL) and generalized inflammation (CRP) were measured in patients with DKD, LN, and in age-matched controls *via* Ella (Protein Simple) according to manufacturer instructions. Briefly, samples were diluted in diluent buffer and 50 *μ*l of the sample was loaded on each well of a CART, and 1 ml of washing buffer was added to the respective well. Samples were then run in triplicates using a microfluidics system proprietary of the Ella Instrument (Protein Simple). Sample analysis was automatically generated utilizing the Simple Plex Runner Software as described in [9].

### 2.3. Statistical and Biomarker Analyses

All samples were run for the expression of all analytes. Samples included in the analysis were only those that were within the lower and upper limit of detection for each analyte. Samples that presented protein levels outside the limit of detection of the assay for each analyte were dropped for such analyte. In addition, outliers were removed prior to further statistical analyses using the Robust regression and outlier removal (ROUT) method with a *Q* set to 1%. Differences between patien groups were tested by one-way ANOVA followed by Tukey's multiple comparison test. Significance was set at *p* < 0.05. Simple Plex data from DKD, LN, and control samples were analyzed utilizing Prism 9 software (GraphPad). Specificity, sensitivity, confidence interval (95%), standard deviation, *p* value, and likelihood ratio was identified through calculation of receiver operating characteristics (ROC) and the total area under the curve (AUC). Additionally, cut-off points and positive and negative predictive values were calculated along with overall assay accuracy. The cut-off point for each analyte was chosen based on the highest likelihood ratio in the sensitivity vs. 1-specificity plot, considering a higher sensitivity than higher specificity values, in order to obtain assays that provide a higher likelihood of reliability for the respective sensitivity for each analyte [24]. In addition, a two-tailed Pearson *r* correlation matrix was carried using Prism 9 software (GraphPad). Sample size in this study was calculated by power analysis with the power set to 0.8 and considering the standard deviation and the difference between the means to obtain a significance of 0.05 for each analyte.

Combination of biomarkers was achieved by fitting a logistic regression model to explain the diagnosis of either DKD or LN followed by calculation of the AUC using RStudio (version 1.2.5033).

ROC curve comparison between analytes was done as described in [25] using the following formula to obtain a critical ratio *Z*:

(1) $$\begin{array}{c} z=\frac{\left(A_{1}-A_{2}\right)}{\sqrt{{{\text{SE}}_{1}}^{2}+{{\text{SE}}_{2}}^{2}-2\text{r}{\text{SE}}_{1}{\text{SE}}_{2}}}. \end{array}$$

And the *p* value was determined using the following formula using Microsoft Excel (version 16.57):

(2) $$\begin{array}{c} =2*\left(1-\text{NORMSDIST}\left(\text{z}\right)\right) \end{array}$$

## 3. Results

### 3.1. ASC and IL-18 Is Elevated in the Serum of Patients with DKD and LN

**Figure 1:**
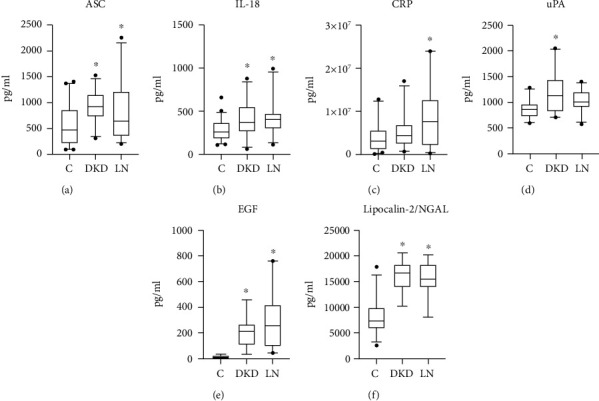
Biomarker protein levels in the serum of patients with DKD and LN. Box and whisker plots showing the protein levels in pg/ml of (a) ASC, (b) IL-18, (c) CRP, (d) uPA, (e) EGF, and (f) Lipocalin-2/NGAL in the blood serum of controls (C), diabetic kidney disease (DKD), and lupus nephritis (LN). Differences between groups were tested *via* one-way ANOVA followed by Tukey's multiple comparison test. Significance was set at *p* < 0.05. ASC: *N* = 43 C, 28 DKD, 32 LN; IL-18: *N* = 43 C, 31 DKD, 32 LN; CRP: *N* = 39 C, 23 DKD, 29 LN; uPA: *N* = 23 C, 20 DKD, 25 LN; EGF: *N* = 17 C, 18 DKD, 24 LN; and NGAL: *N* = 24 C, 14 DKD, 17 LN. Box and whiskers are shown for the 5^th^ and 95^th^ percentile. Dots correspond to data points outside the 5^th^ and 95^th^ percent confidence intervals. ^∗^*p* < 0.05 compared to the control.

The inflammasome has been previously shown to be involved in the pathology of renal diseases [26]. To determine the levels of the inflammasome signaling proteins, ASC and IL-18, serum from DKD and LN patients, were analyzed and compared to age-matched healthy controls for the expression of ASC (Figure 1(a)) and IL-18 (Figure 1(b)) proteins. ASC and IL-18 proteins were shown to be significantly elevated in both DKD and LN patients compared to healthy controls. These results suggest that innate immune inflammatory activity is present in the pathology of both DKD and LN.

### 3.2. Biomarkers of Renal Disease Are Elevated in the Serum of DKD and LN Patients

CRP is a well-known biomarker of general inflammation. Similarly, uPA, EGF, and Lipocalin-2 have demonstrated involvement in the pathology of renal disease [27]. We analyzed serum of patients with DKD and LN for the expression of CRP (Figure 1(c)), uPA (Figure 1(d)), EGF (Figure 1(e)), and Lipocalin-2/NGAL (Figure 1(f)). Levels of CRP were higher in the LN group and not in the DKD group, whereas levels of uPA were higher in the DKD group but not in the LN group. Protein levels of EGF and Lipocalin-2/NGAL were higher in both groups when compared to the control group. Thus indicating that in this cohort of patients, there is a renal dysfunction consistent with the diagnosis of chronic renal diseases.

### 3.3. Biomarkers of DKD

**Figure 2:**
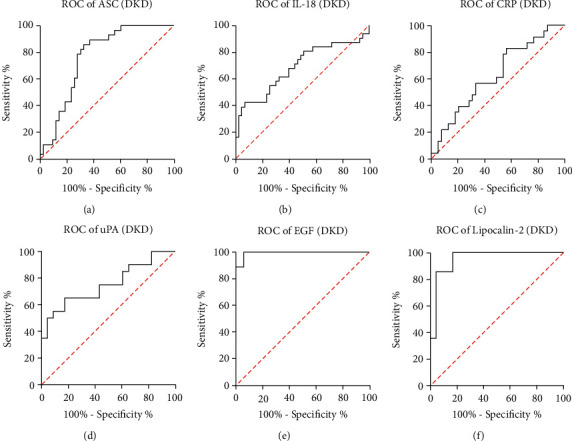
ROC curve of biomarker proteins in patients with DKD. ROC curves indicate the AUC (sensitivity vs 1-specificity) for (a) ASC, (b) IL-18, (c) CRP, (d) uPA, (e) EGF, and (f) Lipocalin-2/NGAL in the serum of patients with DKD. ASC: *N* = 43 C, 28 DKD, 32 LN; IL-18: *N* = 43 C, 31 DKD, 32 LN; CRP: *N* = 39 C, 23 DKD, 29 LN; uPA: *N* = 23 C, 20 DKD, 25 LN; EGF: *N* = 17 C, 18 DKD, 24 LN; and NGAL: *N* = 24 C, 14 DKD, 17 LN.

To assess if the tested proteins could be reliable biomarkers of DKD, the area under the curve (AUC) was calculated for each protein of interest (Table 2). Of the proteins of interest, EGF (Figure 2(e)) and Lipocalin/NGAL (Figure 2(f)) were shown to have the highest AUC, 0.9935 and 0.9554, respectively, followed by ASC, 0.7666 (Figure 2(a)); uPA, 0.7522 (Figure 2(d)); and IL-18, 0.6849 (Figure 2(b)). The AUC for CRP, 0.6254 (Figure 2(c)) was not significant. Sensitivity of Lipocalin-2 was 100%, with an 83% specificity and a cut-off point of 10,187 pg/ml. Similarly, for EGF, sensitivity was 100% with a 94% specificity and a cut-off point of 27.99 pg/ml. With a cut-off point of 632 pg/ml, the sensitivity for ASC was 89% with a 63% specificity (Table 3). These findings indicate that EGF, Lipocalin-2/NGAL, ASC, uPA, and IL-18 can be used as part of a biomarker panel to diagnose DKD.

### 3.4. Biomarkers of LN

**Figure 3:**
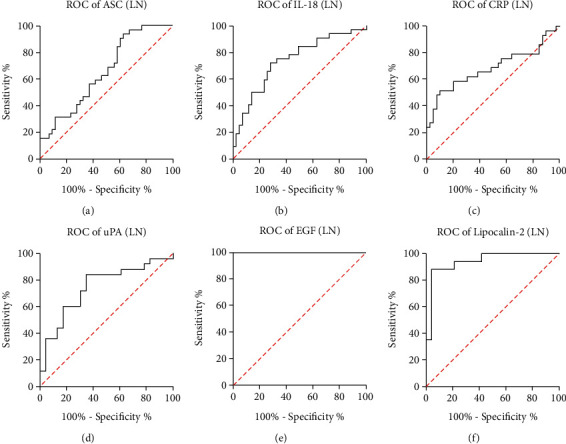
ROC curve of biomarker proteins in patients with LN. ROC curves indicate the AUC (sensitivity vs 1-specificity) for (a) ASC, (b) IL-18, (c) CRP, (d) uPA, (e) EGF, and (f) Lipocalin-2/NGAL in the serum of patients with LN. ASC: *N* = 43 C, 28 DKD, 32 LN; IL-18: *N* = 43 C, 31 DKD, 32 LN; CRP: *N* = 39 C, 23 DKD, 29 LN; uPA: *N* = 23 C, 20 DKD, 25 LN; EGF: *N* = 17 C, 18 DKD, 24 LN; and NGAL: *N* = 24 C, 14 DKD, 17 LN.

To assess if the tested proteins could be reliable biomarkers of LN the area under the curve (AUC) was calculated for each protein of interest (Table 2). Of the proteins of interest, EGF (Figure 3(e)) and Lipocalin/NGAL (Figure 3(f)) were shown to have the highest AUC of 1.00 and 0.9412, respectively. The AUC for ASC was 0.6526 (Figure 3(a)); for uPA, 0.7443 (Figure 3(d)); for IL-18, 0.7384 (Figure 3(b)); and for, CRP 0.6844 (Figure 3(c)). Sensitivity of Lipocalin-2 was 88%, with an 83% specificity and a cut-off point of 10,516 pg/ml. Similarly, for EGF, the sensitivity was 100% with a 100% specificity and a cut-off point of 38.53 pg/ml. For uPA, the cut-off point was 890.3 pg/ml with an 84% sensitivity and a 65% specificity, whereas with a cut-off point of 521.6 pg/ml, the sensitivity for ASC was 63% with a 53% specificity (Table 3). These findings indicate that EGF, NGAL, uPA, IL-18, ASC, and CRP can be used as part of a biomarker panel to diagnose LN.

Furthermore, we compared the ROC curves between all biomarkers analyzed for the cohorts of DKD (Supplementary Table 1) and LN (Supplementary Table 2) patients, and we found that in DKD patients the AUC was significantly different when comparing ASC with EGF (*p* = 2.12*E* − 05), ASC with NGAL (*p* = 0.0009), CRP with EGF (*p* = 0.03), CRP with NGAL (*p* = 2.29*E* − 06), uPA with EGF (*p* = 0.001), and uPA with NGAL (*p* = 0.003) (Supplementary Table 1), indicating that based on the AUC, in DKD, NGAL as a biomarker is consistently different to several of the other biomarkers analyzed. Similarly, when comparing the AUC for biomarkers in LN patients, the significant difference between curves was detected when comparing ASC with EGF (*p* = 3.07*E* − 08), IL-18 with EGF (*p* = 9.45*E* − 06), CRP with EGF (*p* = 7.08*E* − 06) and uPA with EGF (0.0004) (Supplementary Table 2), indicating that in LN based on the AUC, EGF as a biomarker is consistently different to several of the other biomarkers analyzed.

In addition, we combined biomarkers that presented lower AUC values with those present in EGF and NGAL to determine if a combination of biomarkers would increase the AUC value. Accordingly, when combining ASC, IL-18, and uPA, we found that the AUC for the diagnosis of DKD, increased to 0.82, and this value was higher than the AUC obtained for each biomarker alone (0.77, 0.68, and 0.75, respectively). Similarly, for the diagnosis of LN, we combined IL-18 and uPA to obtain an AUC of 0.81 that was also higher than the AUC obtained for each of those two biomarkers alone (0.74).

### 3.5. Correlation between DKD Biomarkers

**Figure 4:**
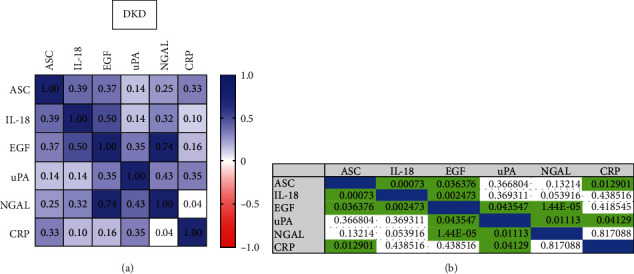
Correlation matrix of biomarker proteins in patients with DKD. (a) Plot shows the correlation between ASC, IL-18, CRP, uPA, EGF, and Lipocalin-2/NGAL in the serum of patients with DKD. (b) *p* values of significance of the Pearson correlation.

To determine the correlation across serum levels of ASC, IL-18, CRP, uPA, EGF, and NGAL, in DKD patients, a Pearson *r* correlation matrix was carried out (Figure 4). The greatest positive correlation was found between NGAL and EGF with an *r* = 0.74 (*p* = 1.44*E* − 05) followed by EGF and IL-18 with an *r* = 0.50 (*p* = 0.002) and NGAL and uPA (*r* = 0.43, *p* = 0.011). The correlation between ASC and IL-18 was 0.39 (*p* = 0.00073) and between ASC and EGF was 0.37 (*p* = 0.036), indicating common trends in the expression between the proteins analyzed in this study in the serum of DKD patients.

### 3.6. Correlation between LN Biomarkers

**Figure 5:**
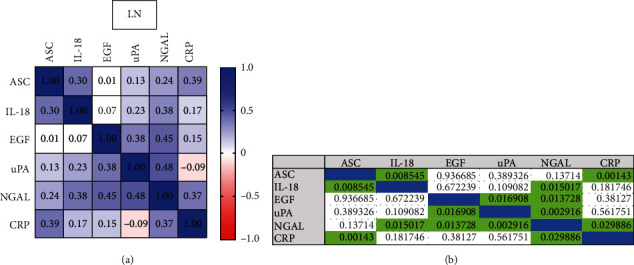
Correlation plot of biomarker proteins in patients with LN. (a) Plot shows the correlation between ASC, IL-18, CRP, uPA, EGF, and Lipocalin-2/NGAL in the serum of patients with LN. (b) *p* values of significance of the Pearson correlation.

To determine the correlation across serum levels of ASC, IL-18, CRP, uPA, EGF, and NGAL in LN patients, a Pearson *r* correlation matrix was carried out (Figure 5). The greatest positive correlation was found between NGAL and uPA with an *r* = 0.48 (*p* = 0.003) followed by EGF and NGAL (*r* = 0.45, *p* = 0.014). The correlation between ASC and CRP was *r* = 0.39 (*p* = 0.001), and between NGAL and CRP the correlation was *r* = 0.37 (*p* = 0.03), indicating common trends in the expression between the analyzed proteins in the serum of LN patients.

## 4. Discussion

DKD and LN are chronic renal disorders associated with kidney damage resulting from inflammation. In this study, we show an array of renal and inflammasome proteins that are not only elevated but are also reliable biomarkers of both disorders. In the serum of DKD and LN patients, we determined an increased expression of the inflammasome scaffolding protein ASC and increased levels of the proinflammatory cytokine IL-18. These findings suggest that inflammation plays a key role in the pathologies of both disorders. Studies have suggested that inflammasome activation is associated with the different pathological manifestations of chronic kidney diseases. For instance, proteinuria has been suggested to act as a NLRP3 activator, while calcium carbonate crystal accumulation, a contributor to fibrosis, was also implicated in NLRP3 activation and tubule inflammation [8].

In DKD, studies have suggested that resulting damage to nephrons and increased albumin excretion are the result of elevated levels in the downstream inflammasome-activated cytokines IL-1*β* and IL-18 [10]. The NLRP3 inflammasome has also been suggested to play a role in the pathology of LN. Accordingly, kidney damage was limited in experiments blocking NLRP3, caspase 1, and IL-18 activation in mouse models of LN [28–31]. Our study supports these findings in humans in that levels of ASC and IL-18 expressions were shown to be significantly elevated in both DKD and LN patients.

In addition to inflammasome activity, our study investigated the levels of other proteins associated with kidney disease and renal function. CRP is a well-established indicator of generalized inflammatory activity and tissue damage [32]. Elevated CRP has been demonstrated in patients with chronic kidney disease, and it has also been implicated in kidney ischemia/reperfusion injury [27, 32]. Here, CRP was elevated in patients with LN but not in DKD. This result is not surprising when considering the different etiologies of the two disorders. Elevated CRP is expected in LN given its role in the complement cascade, and the very close relationship between SLE and the complement system [4, 32].

uPA is produced by tubular epithelial cells, macrophages, and fibroblasts. It has been linked to podocyte function, with increased uAP expression associated with podocyte loss, degradation of renal filtration, and increased proteinuria [33]. It is also thought to play a role in preventing kidney stone formation [34]. In our study, uPA was increased in DKD but not in LN patients, which is consistent with findings in other studies [35].

Serum EGF and Lipocalin-2/NGAL were elevated in DKD and LN. EGF is expressed by cells within the ascending limb of the loop of Henle and the distal tubules and has been shown to have differing effects on renal disease pathology [36]. Lipocalin-2/NGAL is an iron-carrying protein that is expressed by tubular epithelial cells following acute kidney injury and chronic kidney disease [3]. In agreement with our results, other studies have seen increased NGAL expression in patients with LN and DKD [4, 27].

The usefulness of a biomarker is calculated from the ROC to obtain the AUC. The closer to 1.0 or 100%, the better the biomarker. In addition, the ROC provides the sensitivity and a specificity for different cut-off points and associated likelihood ratios to provide the reliability of assaying for a particular biomarker. Accordingly, a likelihood ratio above 1 is consistent with a reliable assay, and the higher this value, the more reliability there is. Moreover, a biomarker is also characterized by a PPV and a NPV which are affected by the prevalence of the disease within the cohort studied, and ultimately, one calculates the accuracy of each biomarker to diagnose the disease. Therefore, in this study, to assess the reliability of the above proteins as potential biomarkers for DKD and/or LN, we determined the ROC to obtain the AUC as well as the sensitivity, specificity, PPV, and NPV of all 6 analytes in this study. Of the analytes for DKD, EGF and Lipocalin-2/NGAL showed the best potential for use as biomarkers based on AUC of 0.99 for EGF and 0.96 for NGAL, followed by 0.76 for ASC and 0.75 for uPA. For EGF and Lipocalin-2/NGAL with cut-off points of 27.99 pg/ml and 10,187 pg/ml, respectively, the sensitivity was 100%. For EGF, the specificity was 94%, and for NGAL, it was 83%. Moreover, correlation matrices of the potential biomarkers showed the greatest correlation between EGF and Lipocalin-2/NGAL. The cut-off point for ASC was 632 pg/ml with a sensitivity of 89% and a specificity of 63%. Similarly, for LN, EGF presented an AUC of 1.0, followed by 0.94 for NGAL and 0.74 for uPA. The cut-off point for EGF was 38.53 pg/ml with a 100% sensitivity and specificity; for NGAL, the cut-off point was 10.516 pg/ml with a 88% sensitivity and 83% specificity, and for uPA, the cut-off point was 890.3 pg/ml with 84% sensitivity and 65% specificity.

In the serum of DKD patients, in addition to NGAL, EGF, and uPA, the inflammasome protein ASC is a reliable biomarker in DKD. Similarly, in the serum of LN, in addition to NGAL, EGF, and uPA, the proinflammatory cytokine IL-18 also presented an AUC consistent with a reliable biomarker of LN in serum. In LN, correlation matrices showed the greatest correlation between NGAL and uPA followed by NGAL and EGF. In fact, NGAL showed significant positive correlations with IL-18, EGF, uPA, and CRP. Thus, these findings point to Lipocalin-2/NGAL as a key biomarker in LN, whereas EGF showed significant positive correlations with ASC, IL-18, uPA, and NGAL, suggesting that EGF is a key biomarker in DKD.

Moreover, it is important to identify theragnostic biomarkers that can provide information regarding response to treatment. The involvement of the inflammasome in renal disease offers a viable therapeutic option to treat such diseases. Thus, we propose that ASC and IL-18 can be used as theragnostic biomarkers of therapies targeting the inflammasome in kidney diseases [37].

Moreover, in the DKD cohort, we performed a linear regression analysis to identify the effects of the biomarkers tested on this study on the eGFR. However, we did not detect a statistically significant contribution of any of these biomarkers to eGFR (data not shown), suggesting that the levels of these protein do not affect disease severity, but instead provide information about the inflammatory response associated with the disease. Similarly, a linear regression was carried out to determine the association between the different classifications of LN and these biomarkers (data not shown). However, no statistical significance was found either. Taken together, these data suggest that the proteins ASC, IL-18, uPA, EGF, NGAL, and CRP contribute significantly to the inflammatory response in renal diseases. However, these proteins do not provide specific clinical information pertaining to the individual pathologies of DKD and LN. Furthermore, future studies should aim at correlating disease severity using individual clinical parameters beyond disease severity to determine whether the potential biomarkers identified in this study can also be used as prognostic biomarkers. In addition, future studies should evaluate the effects of anti-inflammatory therapies in the population of DKD and LN patients to identify whether these proteins can be used as theranostic biomarkers.

In this study, the DKD cohort of patients consisted primarily of type II diabetes patients. Importantly, regardless of the type of diabetes, patients used in the DKD cohort presented kidney disease, suggesting that the findings in this project are more likely related to kidney disease due to diabetes than to the type of diabetes. Future studies are needed to compare the levels of these proteins between patients with type I *vs.* type II diabetes to differentiate whether type I and type II diabetes have different effects on the expression of ASC, IL-18, CRP, uPA, EGF, and NGAL.

Taken together, our results show that some of the most common proteins associated with kidney disease are reliable biomarkers for DKD and LN. Of these proteins, NGAL was the most reliable indicator in both DKD and LN. In addition, ASC is also a good biomarker in DKD. Further studies will focus on identifying how these biomarkers may change as DKD and LN pathology progresses, and whether they prove to be targets for developing therapies to limit DKD or LN progression. In conclusion, EGF and Lipocalin-2/NGAL proteins specifically associated with renal inflammation are reliable biomarkers for assessing chronic kidney disorders, and should be considered in studies looking at DKD and LN disease progression.

## Figures and Tables

**Table 1:** Patients with DKD and lupus nephritis used in the study.

DKD patients	31
LN patients	32

Sex (DKD)	Males (55%)–female (45%)
	17 - 14

Sex (LN)	Males (25%)–female (72%)–unspecified (3%)
	8–23–1

Race (DKD)	
Caucasian	17 (55%)
African	10 (32%)
Other	4 (13%)

Race (LN)	
Caucasian	17 (53%)
African	11 (34%)
Other	4 (13%)

Age range (DKD)	
Range	21 - 90
Median	62
Mean	63.32

Age range (LN)	
Range	19 - 75
Median	43.5
Mean	44.91

Comorbidities (DKD)	
HTN	23 (74%)
Hypercholesterolemia	6 (19%)
Hypothyroidism	8 (26%)
GERD	7 (23%)
CAD	7 (23%)
Diabetes type I	2 (6%)
Diabetes type II	21 (68%)
Diabetes (type unspecified)	8 (26%)

Comorbidities (LN)	
HTN	6 (19%)
GERD	2 (6%)
CAD	3 (9%)

Disease stage (DKD)	
2	1 (3%)
3	7 (22%)
4	2 (7%)
5	2 (7%)
Unspecified	19 (61%)

Disease stage (LN)	
1	4 (12%)
3	6 (19%)
4	5 (16%)
5	2 (6%)
Unspecified	15 (47%)

HTN: hypertension; GERD: gastroesophageal reflux disease; CAD: coronary artery disease.

**(a):** 

Control vs. DKD				
Biomarker	AUC	STD. error	95% C.I.	*p* value
ASC	0.7666	0.05595	0.6570 to 0.8763	0.0002
IL-18	0.6849	0.06612	0.5553 to 0.8145	0.0069
CRP	0.6254	0.07273	0.4829 to 0.7680	0.1011
uPA	0.7522	0.07668	0.6019 to 0.9025	0.0047
EGF	0.9935	0.00877	0.9763 to 1.000	<0.0001
Lipocalin-2/NGAL	0.9554	0.03177	0.8931 to 1.000	<0.0001

**(b):** 

Control vs. LN				
Biomarker	AUC	STD. error	95% C.I.	*p* value
ASC	0.6526	0.06274	0.5296 to 0.7756	0.0245
IL-18	0.7384	0.05906	0.6226 to 0.8541	0.0004
CRP	0.6844	0.07027	0.5466 to 0.8221	0.0097
uPA	0.7443	0.07279	0.6017 to 0.8870	0.0037
EGF	1.000	0.000	1.000 to 1.000	<0.0001
Lipocalin-2/NGAL	0.9412	0.3714	0.8684 to 1.000	<0.0001

**(c):** 

DKD vs. LN				
Biomarker	AUC	STD. error	95% C.I.	*p* value
ASC	0.6373	0.07508	0.4901 to 0.7844	0.0684
IL-18	0.5222	0.07501	0.3752 to 0.6692	0.7623
CRP	0.5997	0.07994	0.4430 to 0.7564	0.2205
uPA	0.5980	0.09194	0.4178 to 0.7782	0.2630
EGF	0.5856	0.08860	0.4120 to 0.7593	0.3470
Lipocalin-2/NGAL	0.5546	0.1057	0.3474 to 0.7618	0.6058

**(a):** 

Control vs. DKD							
Biomarker	Cut-off point (pg/ml)	Sensitivity (%)	Specificity (%)	PPV (%)	NPV (%)	Likelihood ratio	Accuracy (%)
ASC	>632	89	63	61	90	2.400	73
IL-18	>268.7	74	53	53	74	1.595	62
CRP	> 2,984,567	61	51	42	69	1.249	55
uPA	> 875	70	57	58	68	1.610	63
EGF	> 27.99	100	94	95	100	17.00	97
Lipocalin-2	> 10,187	100	83	78	100	6.00	89

**(b):** 

Control vs. LN							
Biomarker	Cut-off point (pg/ml)	Sensitivity (%)	Specificity (%)	PPV (%)	NPV (%)	Likelihood ratio	Accuracy (%)
ASC	> 521.6	63	53	50	66	1.344	57
IL-18	> 278.9	78	58	58	78	1.866	67
CRP	> 3,768,714	66	62	56	71	1.703	63
uPA	> 890.3	84	65	72	79	2.415	75
EGF	> 38.53	100	100	100	100	—	100
Lipocalin-2	> 10,516	88	83	79	91	5.294	85

## Data Availability

The data presented in this study are available on request from the corresponding author.

## Supplementary Materials

Supplementary Materials Supplementary Table 1: DKD: ROC curve comparison. Supplementary Table 2: LN: ROC curve comparison.
