# Supplementary material for: Renal and Inflammatory Proteins as Biomarkers of Diabetic Kidney Disease and Lupus Nephritis
Source: Oxid Med Cell Longev. 2022 Mar 20;2022:5631099. doi: 10.1155/2022/5631099 (PMC8958067; doi:10.1155/2022/5631099)
Supplement: Supplementary Materials — Supplementary Table 1: DKD: ROC curve comparison. Supplementary Table 2: LN: ROC curve comparison. [file 5631099.f1.docx]

**Supplementary Table 1. DKD: ROC curve comparison**

| **ASC** | **ASC vs IL-18** | **ASC vs CRP** | **ASC vs uPA** | **ASC vs EGF** | **ASC vs NGAL** |
| --- | --- | --- | --- | --- | --- |
|  | 0.22757644 | 0.061623855 | 0.870223651 | 2.12463E-05 | 0.000915715 |
| **IL-18** | **IL-18 vs CRP** | **IL-18 vs uPA** | **IL-18 vs EGF** | **IL-18 vs NGAL** |  |
|  | 0.599070439 | 0.631817206 | 0.533337664 | 0.361817307 |  |
| **CRP** | **CRP vs uPA** | **CRP vs EGF** | **CRP vs NGAL** |  |  |
|  | 0.728795294 | 0.028209707 | 2.29457E-06 |  |  |
| **uPA** | **uPA vs EGF** | **uPA vs NGAL** |  |  |  |
|  | 0.001125391 | 0.003337255 |  |  |  |
| **EGF** | **EGF vs NGAL** |  |  |  |  |
|  | 0.141869378 |  |  |  |  |

**Supplementary Table 2. LN: ROC curve comparison**

| **ASC** | **ASC vs IL-18** | **ASC vs CRP** | **ASC vs uPA** | **ASC vs EGF** | **ASC vs NGAL** |
| --- | --- | --- | --- | --- | --- |
|  | 0.23282756 | 0.66614537 | 0.30708732 | 3.0746E-08 | 0.42475211 |
| **IL-18** | **IL-18 vs CRP** | **IL-18 vs uPA** | **IL-18 vs EGF** | **IL-18 vs NGAL** |  |
|  | 0.5193315 | 0.94284675 | 9.4498E-06 | 0.56603772 |  |
| **CRP** | **CRP vs uPA** | **CRP vs EGF** | **CRP vs NGAL** |  |  |
|  | 0.57082473 | 7.0807E-06 | 0.46529259 |  |  |
| **uPA** | **uPA vs EGF** | **uPA vs NGAL** |  |  |  |
|  | 0.00044334 | 0.56570284 |  |  |  |
| **EGF** | **EGF vs NGAL** |  |  |  |  |
|  | 0.87420475 |  |  |  |  |
